# Medial Prefrontal Cortex Stimulation Reduces Retrieval-Induced Forgetting via Fronto-parietal Beta Desynchronization

**DOI:** 10.1523/JNEUROSCI.0189-24.2024

**Published:** 2024-08-15

**Authors:** Ahsan Khan, Chun Hang Eden Ti, Kai Yuan, Maite Crespo Garcia, Michael C. Anderson, Raymond Kai-Yu Tong

**Affiliations:** ^1^Department of Biomedical Engineering, The Chinese University of Hong Kong, Hong Kong, China; ^2^MRC Cognition and Brain Sciences Unit, University of Cambridge, Cambridge CB2 7EF, United Kingdom

**Keywords:** beta desynchronization, inhibitory control, medial prefrontal cortex, memory retrieval, retrieval-induced forgetting, tDCS

## Abstract

The act of recalling memories can paradoxically lead to the forgetting of other associated memories, a phenomenon known as retrieval-induced forgetting (RIF). Inhibitory control mechanisms, primarily mediated by the prefrontal cortex, are thought to contribute to RIF. In this study, we examined whether stimulating the medial prefrontal cortex (mPFC) with transcranial direct current stimulation modulates RIF and investigated the associated electrophysiological correlates. In a randomized study, 50 participants (27 males and 23 females) received either real or sham stimulation before performing retrieval practice on target memories. After retrieval practice, a final memory test to assess RIF was administered. We found that stimulation selectively increased the retrieval accuracy of competing memories, thereby decreasing RIF, while the retrieval accuracy of target memories remained unchanged. The reduction in RIF was associated with a more pronounced beta desynchronization within the left dorsolateral prefrontal cortex (left-DLPFC), in an early time window (<500 ms) after cue onset during retrieval practice. This led to a stronger beta desynchronization within the parietal cortex in a later time window, an established marker for successful memory retrieval. Together, our results establish the causal involvement of the mPFC in actively suppressing competing memories and demonstrate that while forgetting arises as a consequence of retrieving specific memories, these two processes are functionally independent. Our findings suggest that stimulation potentially disrupted inhibitory control processes, as evidenced by reduced RIF and stronger beta desynchronization in fronto-parietal brain regions during memory retrieval, although further research is needed to elucidate the specific mechanisms underlying this effect.

## Significance Statement

Retrieval can induce forgetting of competing memories, a phenomenon known as retrieval-induced forgetting (RIF). Inhibitory control mechanisms, primarily mediated by the frontal cortex, are thought to contribute to RIF. In this study, we modulated a key region [medial prefrontal cortex (mPFC)] involved in this process using brain stimulation to investigate its influence on RIF and its electrophysiological correlates. Stimulation of mPFC led to an increased retrieval of non-target memories and reduced RIF. The reduction in RIF was accompanied by stronger beta desynchronization in the fronto-parietal brain regions, with beta desynchronization in the left-dorsolateral prefrontal cortex predicting the extent of reduction in RIF.

## Introduction

The act of recalling a memory may not only facilitate the retrieval of intended information, reflecting a memory facilitation effect (FAC) of target memories but can also induce forgetting of competing non-target memories, a phenomenon referred to as retrieval-induced forgetting (RIF; Anderson et al., 1994; see Anderson, 2003; Anderson and Hulbert, 2020; Marsh and Anderson, 2022 for reviews). Despite extensive research efforts, the underlying psychological and neurobiological mechanisms of RIF remain only partially understood. This study investigates these mechanisms by applying stimulation to a key region within the episodic memory network, namely, the medial prefrontal cortex (mPFC), and examining its impact on behavioral and electrophysiological indicators of RIF.

Inhibitory control processes are thought to contribute to RIF (Anderson, 2003; Storm and Levy, 2012; Murayama et al., 2014; Anderson and Hulbert, 2020; Marsh and Anderson, 2022). According to the inhibitory model, two processes contribute to selectively retrieving a target trace in the face of competition from alternative memories during retrieval practice. First, when retrieval cues appear, a retrieval process automatically activates all traces in memory associated to those cues. Second, in response to this diverse activation, an inhibitory control mechanism is recruited to resolve interference from non-target traces by inhibiting those competitors, facilitating target retrieval. An alternative explanation instead suggests that retrieving target memories strengthens those retrieved items, and this dominance causes them to block access to competitors on the final test (for a review of alternatives, see Anderson et al., 1994; Anderson, 2003). Thus, the inhibition and blocking views diverge on the relationship between FAC and RIF: whereas the non-inhibitory model proposes a direct link between their strengths, the inhibitory model posits them as functionally independent.

A large body of research on inhibitory control in attention involving the Stroop, Flanker, and Simon tasks suggests that the anterior cingulate cortex (ACC) that sits in the mPFC detects the conflict and upregulates lateral prefrontal cortex (LPFC) activity to resolve it (Hanslmayr et al., 2008; Kim et al., 2014; Crespo-García et al., 2022). This same conflict detection mechanism is thought to detect competition during memory retrieval (Kuhl et al., 2007; Crespo-García et al., 2022) and to dynamically adjust as conflict is reduced. For example, electrophysiological studies indicate that (1) increased midfrontal theta activity is associated with control demands required during retrieval of memories (Staudigl et al., 2010) and (2) decreases in mid-frontal theta power over repeated retrievals predict greater RIF, reflecting a conflict reduction benefit (Hanslmayr et al., 2010). In addition, beta band activity in the medial and lateral prefrontal regions has also been associated with inhibitory control. Studies have shown that successfully suppressing a thought during retrieval (Castiglione et al., 2019), directed forgetting at encoding (Hubbard and Sahakyan, 2023), or even inhibiting a saccade (Hwang et al., 2014) all lead to increased beta band activity in the LPFC regions. Together, these studies point to the involvement of mPFC and LPFC regions in inhibitory control, with changes in theta and beta band activity frequently serving as markers of this involvement.

In this study, we investigated whether stimulating the mPFC using tDCS can modulate RIF. Additionally, we studied the electrophysiological correlates associated with this modulation. Following the study phase, we administered 2 mA direct current stimulation to the mPFC for 15 min while participants rested. Immediately after stimulation, participants engaged in retrieval practice tasks while their brain activity was monitored using electroencephalography (EEG). We posited that by stimulating the mPFC before engaging in retrieval practice, participants’ ability to detect and inhibit interfering memories during the retrieval practice trials would be selectively modulated without any influence on performance in retrieval practice trials. If so, stimulation should reduce indices of RIF during the later test phase but have little effect on FAC. In addition, the modulation in RIF would be accompanied by corresponding changes in the brain's neural activity patterns, particularly in theta and beta band activity.

## Materials and Methods

### Participants

Fifty participants (23 females, mean ± SD age, 21.4 ± 2.0 years) were recruited for the study from the student pool of the Chinese University of Hong Kong. All participants were right-handed, with normal or corrected-to-normal vision, and no reported history of psychiatric or neurological illness. Participants were briefed on the protocol, signed a written consent form, and were randomly assigned to stimulation and sham groups with no difference in age (*t* = 0.342; *p* = 0.245). All participants were paid for their participation. The study was conducted in accordance with the Helsinki declaration and was approved by the ethics committee of the Chinese University of Hong Kong.

### Retrieval practice paradigm

Typically, the retrieval practice paradigm is used to study RIF, which consists of three phases (Anderson et al., 1994). In an initial study phase, participants learn category-exemplar pairs from several categories for a later memory test. In a subsequent retrieval practice phase, half of the items from a subset of the studied categories (hereinafter referred to as “practiced categories”) are retrieved repeatedly. A final test phase then occurs in which participants are tested on all of the studied items. Memory for the practiced items is enhanced compared with non-practiced items from non-practiced categories (i.e., control items), reflecting a memory facilitation effect (FAC). Critically, however, final recall performance for non-practiced items from practiced categories—items that would have directly competed with practiced items during retrieval practice trials is reduced compared with recall performance for control items. The tendency for retrieval practice to impair retention of competing items reflects the RIF phenomenon, a form of active forgetting.

The RIF paradigm employed in the study consisted of 24 categories, each containing eight exemplar words in the Chinese-Mandarin language. Each category included four strong exemplars that were relatively easy to recall and four weak exemplars that were more challenging to remember. All exemplars were Chinese double-character words with clear meanings and were selected from word database of Chinese speakers (Liu, 2013). In the study phase, participants studied all the items in category-exemplar word pair format (e.g., Fruit-Apple). In the retrieval practice phase, participants performed retrieval practice on only the 4 weak exemplars from half (12) of the categories, with each of these items practiced three times. Retrieving memories is thought to induce forgetting in part because inhibitory control is triggered to resolve competition between competing and non-competing memories. Thus, items that compete for retrieval with a target trace should be inhibited more than should weaker non-interfering competitors. Because strong category exemplars, such as (Fruit-Apple) are highly accessible, they are thought to pose greater competition than do weak exemplars, such as (Fruit-Kiwi), necessitating more inhibitory control, yielding larger RIF during the test phase. Because this prediction has been confirmed by numerous studies (see Murayama et al., 2014 for a meta-analysis), we used weak exemplars as items to be retrieved during retrieval practice, and strong exemplars as items to be forgotten.

Retrieval practice trials presented participants with the category name and a distinctive cue for the appropriate exemplar and required participants to recall the studied item that fit those cues (e.g., Fruit Ap___). After administering a short distractor task, we then tested the category-exemplar pairs using a category-plus stem cued recall test (e.g., Fruit A___). Thus, 192 exemplars were divided into four types, including the practiced weak items from practiced categories (RP+ items), the non-practiced strong items from practiced categories (RP− items), the weak non-practiced items from non-practiced categories (NRP+ items), and the strong non-practiced items from non-practiced categories (NRP− items). NRP+ and NRP− trials served as control items for RP+ and RP− items, respectively.

### Experimental design

**Figure 1.**
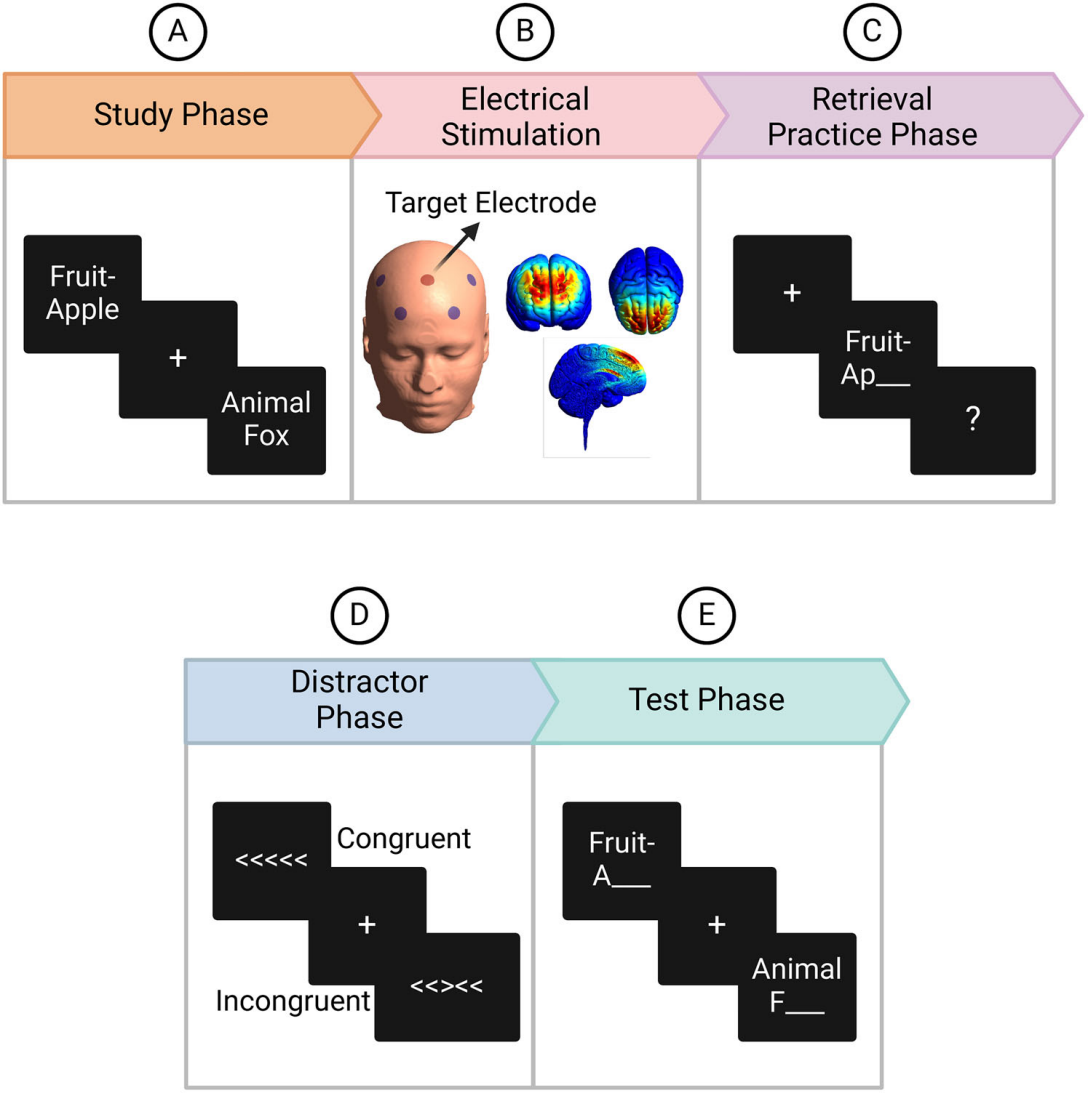
Experimental Design. ***A***, During the study phase, participants were instructed to study the items presented on the screen and were told that they would be tested later on those items. ***B***, Following the study phase, stimulation was performed for 15 min, during which participants rested. Stimulation electrodes were placed in a ring configuration based on the 10–20 EEG electrode placement method. The target electrode was placed at the Fz location, whereas four return electrodes were placed at AF3, AF4, FC3, and FC4. The simulation generated using SimNIBS is shown. ***C***, Immediately after stimulation, retrieval practice was performed in which participants recalled some of the exemplars (RP+ items) when the category cue associated with the exemplar stem appeared on the screen. Participants performed three retrieval practices for each practiced item. ***D***, Post-stimulation measurements then continued with a distractor phase lasting for 5 min followed by a final memory test phase (***E***) during which memory for all the studied items was tested. Created with BioRender.com.

The experiment was structured into five main parts, including the study phase, electrical stimulation, retrieval practice phase, distractor phase, and test phase appearing in the order outlined in Figure 1. Further details about each of the parts are provided below.

#### Study phase

During the study phase, participants studied all the category-exemplar word pairs and were instructed to remember them for a later recall. We divided the pairs into eight blocks, and each block consisted of 24 exemplars, one from each of the 24 categories. We further divided exemplars in each block into six sub-blocks with four exemplars in each (RP+, RP−, NRP+, NRP−) and exemplars within each sub-block alternated between strong and weak exemplars. We presented a filler item after each block and two buffer items at the start and the end of the experiment to control for primacy and recency effects. Trials appeared on the screen for 3 s each, separated by fixation cross for 1.5–2 s, with the whole phase lasting ∼18 min. Figure 1*A* shows the study phase presentation procedure.

#### Electrical stimulation

We stimulated mPFC with a high-definition transcranial direct current stimulator (HD-tDCS, DC-Stimulator, Soterix Medical). We implanted stimulation electrodes in the EEG cap in a ring configuration. We placed the target (anode) electrode on the Fz location whereas we placed return electrodes (cathodes) at AF3, AF4, FC3, and FC4 locations based on 10–20 systems as shown in Figure 1*B*. This configuration aimed to deliver anodal stimulation, a protocol known to induce excitation in the targeted brain region. Research has indicated that anodal stimulation of mPFC elicits more robust effects on inhibitory control in attention task as compared with cathodal stimulation, which aims to inhibit the target brain region (To et al., 2018). For the stimulation group, we administered a direct current of 2 mA for 15 min; in contrast, for the sham group, the current ramped up to 2 mA in the first 30 s, ramped down to 0 in the next 30 s, with no stimulation provided during the remaining 14 min, and the retrieval task was performed right after. This form of stimulation is referred to as “offline stimulation.” We applied a conductive gel to keep the impedance below 15 kOhm. The stimulation intensity and timing were adopted from previous studies in which 15 min of stimulation given to MPC resulted in modulation of its activity (Khan et al., 2020, 2022). We anticipated the effects of stimulation to last throughout the memory retrieval task, which occurred immediately following the stimulation period and took ∼20 min to complete. This was based on previous research on motor cortex excitability, where 20 min anodal stimulation effects lasted up to 30 min poststimulation (Bashir et al., 2019). We expected the stimulation effect to gradually weaken until the test phase which was administered ∼30 min after the stimulation. The parameters used for stimulation were within the safety limits (Bikson et al., 2009).

#### Retrieval practice phase

We administered the retrieval practice phase after stimulating the mPFC for 15 min. We collected EEG data during this phase. Participants practiced 48 RP+ items, 4 from each of the 12 independent categories. We divided trials into four randomly ordered blocks, each with 12 randomly ordered exemplars, one from each category, with a filler item appearing after each block.

We repeated retrieval practice three times, each separated by a 30 s break. On each retrieval practice trial, cues appeared on the screen for 2 s, and participants were instructed to recall the associated exemplar that they studied during the study phase and speak the correct answer out loud only when “?” appeared on the screen. Figure 1*C* illustrates a typical trial sequence for the retrieval practice task. The experimenter manually recorded whether each participant's response was “accurate,” “not accurate,”, or “not responded”. Each of the three repetitions of the retrieval practice list lasted ∼6 min.

#### Distractor phase

We conducted a 5 min flanker task to keep participants occupied during the distractor phase (Verbruggen et al., 2006). Five arrows appeared in the center of the screen. On congruent trials, all the arrows pointed in the same direction (left or right), whereas on incongruent trials, the central arrow pointed in the opposite direction to the surrounding flankers, as shown in Figure 1*D*. We asked participants to respond to the central arrow by pressing the left or right key to indicate the arrow's direction as quickly and accurately as possible. Trials remained on the screen until the participant pressed the button. A fixation cross lasting for 1.5–2 s appeared after each trial.

#### Test phase

We tested participants on all 192 items in the final test phase. We conducted the test phase ∼30 min after stimulation concluded, anticipating that any influence from the stimulation would have ended by that point. We tested each of the 24 categories, with each category having its own block, and all RP− or NRP− items for a given category tested before all of the RP+ or NRP+ items. We ordered the categories to match the mean position of practiced and non-practiced categories in the test list equating output interference effects. Each test trial appeared on the screen for 3 s. We asked participants to speak the correct answer to the cues aloud whenever the cue appeared on the screen. Figure 1*E* illustrates a representative trial sequence for the final test task. The experimenter manually recorded whether the response was “accurate,” “not accurate,” or “not responded.” The test phase lasted ∼18 min.

### Behavioral data analysis

To test for differences in the percentage of correctly recalled items during the retrieval practice phase, we analyzed performance with a repeated-measures ANOVA using a mixed factorial design with group (stim vs sham) as a between-subject variable and retrieval practice repetition (R1 vs R2 vs R3) as a within-subject variable. Given a significant main effect or interaction effect, we conducted pairwise comparisons.

To test for the occurrence of RIF on the final test and its modulation by stimulation, we conducted a mixed factorial ANOVA on the percentage of items correctly recalled with group (stim vs sham) as a between-subject variable and item type (RP− vs NRP−) as a within-subject variable. We performed a similar analysis to test the impact of stimulation on FAC with group (stim vs sham) as a between-subject variable and item type (RP+ vs NRP+) as within-subject variables. Post hoc comparisons were performed if any main effect or interaction arose. Finally, correlation analysis was conducted between FAC and RIF for each of the two groups using robust correlation toolbox (Pernet et al., 2012).

### Electroencephalography

#### Data acquisition

EEG was acquired using a 64-channel Neuroscan system (SynAmps2, Neuroscan) at 1 kHz sampling frequency with electrodes placed according to the standard 10–20 system. Two pairs of bilateral Electrooculogram (EOG) electrodes were used to collect vertical and horizontal EOG signals. A conductive gel was used to keep the impedance below 5 kOhm. An electrode between Cz and CPz was used as a reference electrode, and AFz acted as the ground electrode.

#### Preprocessing

Preprocessing was performed using MATLAB R2021b [MathWorks (2021)], EEGLab toolbox (Delorme and Makeig, 2004), and Neuroscan Curry (Neuroscan, 2008). The raw data was downsampled to 250 Hz and was bandpass filtered to 1–30 Hz. Before further analysis, the stimulation electrodes were removed, and data was rereferenced using a common average reference. Vertical and horizontal EOG signals were removed from the EEG data using covariance analysis to suppress covarying signals in each EEG channel (Semlitsch et al., 1986). The data was then segmented into 3,000 ms epochs, which included a 2,000 ms poststimulus window, and an additional 1,000 ms prestimulus window. We further cleaned the data by removing sections with unusual electrical activity. Specifically, epochs were marked if the voltage spike was >100 µV either positively or negatively or if the probability distribution exceeded 5 standard deviations from the mean value, either on a single electrode or across all electrodes. We double-checked this by visually inspecting the data before rejecting. After removing trials, the number of trials analyzed for the stimulation group was as follows: R1 (mean, 44.52 trials; SD, 4.574 trials), R2 (mean, 44.84 trials; SD, 4.069 trials), and R3 (mean, 44.16 trials; SD, 6.517 trials). The sham group had comparable results with analyzed trials in R1 (mean, 42.8 trials; SD, 7.141 trials), R2 (mean, 44.96 trials; SD, 3.599 trials), and R3 (mean, 43.12 trials; SD, 4.737 trials).

#### Time–frequency analysis

Time–frequency representations (TFRs) were computed using complex Morlet wavelets ranging from 3 to 10 cycles with the Gaussian width defined as full-width at half-maximum (FWHM) in the frequency domain (Cohen, 2019). TFRs were computed within the 1,500 ms epoch with a prestimulus baseline of 500 and 1,000 ms of poststimulus period. All the trials were averaged, and baseline correction was performed using the prestimulus period. Cluster-based permutation testing was then implemented in FieldTrip (Oostenveld et al., 2011) to identify spatiospectral clusters showing significant group and interaction effects. To estimate the interaction effect between groups for retrieval sessions, we first took a difference for each time point between first and third retrieval sessions (R3–R1) and then subjected this difference to test for group differences (stim vs sham). To assess the group effect, we averaged the data from both R1 and R3 sessions and compared the overall activation patterns between the two groups, while controlling for multiple-comparisons problem. A two-sample *t* test was conducted between the stimulation and sham group for each sample of the channel–time–frequency triplet. Samples with *p* > 0.001 were excluded and the survived samples adjacent to each other were grouped together into clusters. The spatial constraint to include clusters in follow-up analysis was set to a minimum of two neighboring channels. An empirical distribution of the maximum across the sum of *t* values within each cluster was generated by computing the *t* statistics after 1,000 random Monte Carlo permutations. Observed clusters with sum of *t* values having a *p* value of <0.05 from the empirical distribution were considered significant.

#### Source analysis

The specific time–frequency windows used for subsequent source imaging were determined from the significant group and interaction effects observed after permutation analysis on the sensor level data. First, the source model was defined as a 5 mm equally spaced three-dimensional grid and further warped into the standard MNI space. A single-shell head model (Nolte, 2003) was adopted based on a standard T1-weighted MRI and was coregistered to the standard electrode locations. The power in each of the significant cluster was projected onto the source space using a dynamical imaging of coherent sources (DICS) beamformer (Gross et al., 2001). DICS is a beamforming technique that uses the cross-spectral density matrix to estimate the coherence between all pairs of EEG channels. The beamformer then selects signals that are coherent at a specific frequency and multiplies them by the inverse of the lead field matrix to reconstruct the sources of the EEG activity. The power within the baseline period was also projected to the source space, and the event-related desynchronization (ERD) values for specific time of interest were calculated based on the following formula:

$$\mathrm{ER}D_{\mathrm{source}}=10\times \log_{10}\frac{P_{\mathrm{TOI}}}{P_{\mathrm{baseline}}}.$$

All the source analysis procedures were carried out using the FieldTrip toolbox (Oostenveld et al., 2011). Statistical analysis conducted for source-level ERD was similar to permutation. The significance level was set to *p* < 0.01 at the voxel level and further corrected using the Gaussian random field theory at the cluster level.

## Results

### No change in retrieval practice performance

**Figure 2.**
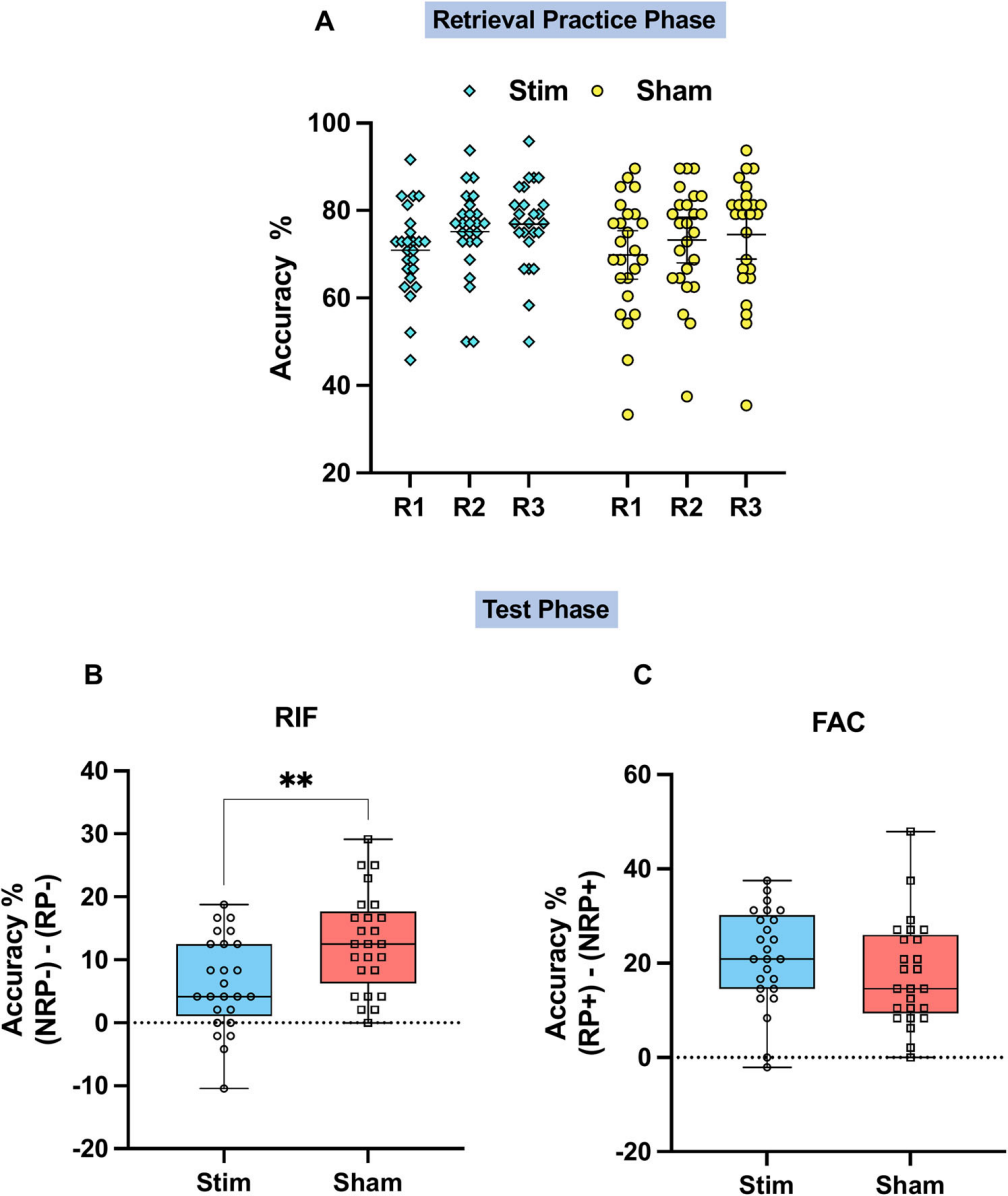
Behavioral results during retrieval and test phase are shown. ***A***, The percentage of items correctly retrieved during three retrieval practice phases (R1, R2, and R3) for the stimulation and sham groups. Accuracy for both groups increased over repetitions, and no significant interaction was observed between groups. ***B***, RIF effects measured by taking the difference between the percentage accuracy of NRP− and RP− trials during the final memory test phase are shown. The stimulation group showed a significantly reduced RIF compared with the sham group (**p < 0.01, ANOVA between groups for RP− and NRP− trials). ***C***, FAC effects measured by taking the difference between the percentage accuracy of RP+ trials and NRP+ trials are shown. FAC was slightly stronger in the stimulation group as compared with the sham group; however, the difference was non-significant. Each dot on the box plot represents an individual participant. The plots were generated using GraphPad Prism (version 10.0; GraphPad Software).

Over the course of the three retrieval practice trials, there was a notable improvement in recall performance, indicating a significant main effect of repetition on retrieval accuracy (*F*_(2,96)_ = 64.14; *p* < 0.001). However, this pattern was consistent for both groups and did not exhibit an interaction between the groups (*F*_(2,96)_ = 0.96; *p* = 0.386), as illustrated in Figure 2*A*. Follow-up paired *t* tests confirmed this pattern, revealing significant increases in retrieval accuracy from R1 to R2 (*t*_(49)_ = 7.73; *p* < 0.001), from R1 to R3 (*t*_(49)_ = 9.65; *p* < 0.001), and from R2 to R3 (*t*_(49)_ = 3.80; *p* < 0.001). The results indicate that the stimulation of the mPFC before retrieval practice did not influence retrieval of the target memories during retrieval practice.

### Selective modulation of RIF

Next we examined behavioral performance during the test phase. To examine stimulation-induced effects on RIF, we conducted a mixed ANOVA with group (stim vs sham) as a between-subjects factor and trial type (RP− vs NRP−) as a within-subjects factor. The results showed a significant main effect for item type (*F*_(1,48)_ = 78.82; *p* < 0.001), demonstrating that the final recall of NRP− items (mean = 69.42; SD = 8.43%) was better than the recall of RP− items (mean = 59.82; SD = 9.55%), across groups. More interestingly, the amount of RIF varied depending on the nature of the stimulation: we observed an interaction of item type with stimulation group (*F*_(1,48)_ = 9.07; *p* = 0.004), and this interaction arose due to a weaker RIF effect in the stimulation group (mean = 6.25; SD = 7.36%) than the sham group (mean = 12.83; SD = 7.81%). Notably, the RIF effect was significant in both the groups with stimulation group (*t*_(24)_ = 4.250; *p* < 0.001) and sham group (*t*_(24)_ = 8.215; *p* < 0.001) showing significant results in the *t* test conducted between RP− and NRP−. The reduction in RIF in the stimulation group was driven mainly by higher recall of RP− items (mean = 62.33; SD = 9.78%), compared with the sham group (mean = 57.33; SD = 8.80%), consistent with the hypothesis that stimulation disrupted the ability to inhibit those items.

In addition, this modulation was specific to RIF: FAC was uninfluenced by stimulation. A mixed ANOVA with group (stim vs sham) as between-subjects variable and item type (RP+ vs NRP+) as a within-subjects variable showed a significant main effect for item type (*F*_(1,48)_ = 165.84; *p* < 0.001). Further analyses revealed that participants recalled significantly more RP+ items (mean = 73.08; SD = 11.96%) than NRP+ items (mean = 53.50; SD = 11.00%), and this effect did not vary across the two groups (*F*_(1,48)_ = 1.32; *p* = 0.256). The percentages of items correctly recalled for each of the item types are shown in Table 1 and differences in RIF and FAC are shown in Figure 2*B*,*C*, respectively. Participants in the stimulation group also showed slightly improved memory retrieval for practiced items (RP+). While this improvement was not statistically significant (*t*_(48)_ = 1.136; *p* = 0.261), it raises the possibility that stimulation might have influenced some general memory mechanisms related to only practiced categories, not necessarily specific to the inhibitory control. Assuming a general improvement in practiced categories, the effect of stimulation should have theoretically impacted both (RP+) and (RP−) items proportionally. This would have resulted in a positive correlation between the FAC and RIF measures within the stimulation group. In order to test this hypothesis, we correlated the amount of FAC and the amount of RIF observed for a given participant and we found no significant correlations for either the stimulation (skipped Pearson *r*_(23)_ = 0.107; CI = [−0.346, 0.502]) or the sham groups (skipped Pearson *r*_(23)_ = −0.129; CI = [−0.484, 0.297]). This lack of correlation provides evidence that stimulation influenced cognitive processes that were specific to RIF and had no general influence on retrieval accuracy of memories from the practiced categories.

**Table 1.** The percentage accuracy (mean and standard deviation) of memory recall for all the trial types (RP+, NRP+, RP−, NRP−) in the stimulation and sham groups during the test phase

Groups	RP+	NRP+	RP−	NRP−
Stim (mean ± SD)	75 ± 10.69	53.67 ± 10.01	**62.33 ± 9.79**	68.67 ± 9.80
Sham (mean ± SD)	71.17 ± 13.05	53.33 ± 12.10	**57.33 ± 8.80**	70.17 ± 6.93

The values highlighted in bold reflect the trial types that significantly differed between stimulation and sham groups.

Consistent with our hypothesis, stimulation of the mPFC selectively reduced RIF effect in the stimulated group. Interestingly, this modulation was specific to RIF, with no change observed in FAC. This finding provides further support for the independence of RIF and FAC, as proposed by the inhibitory model, and suggests that mPFC stimulation may have influenced inhibitory control over memory.

### Beta desynchronization in DLPFC indexes modulation of processes that cause RIF

To examine the neural correlates of processes that modulate RIF and identify potential differences between stimulation and sham groups, we employed a non-parametric cluster-based permutation approach on EEG data collected during the retrieval practice phase. This method is particularly well suited for analyzing EEG data when investigating localized patterns of activation and due to its ability to handle multiple comparisons without assuming a specific distribution for the data. We were particularly interested in examining how neural activity is modulated when the retrieval cue is encountered for the first time during retrieval practice sessions and how this modulation progresses until the final retrieval session. Thus, the analysis was restricted to only the first and third retrieval sessions. For interaction effects, we subjected the difference between the first and third retrieval sessions (R3 − R1) to test for group (stim vs sham) differences, and to assess the group effect, we combined the activation data from both retrieval sessions (R1 + R3) and compared the overall activation patterns between the two groups.

**Figure 3.**
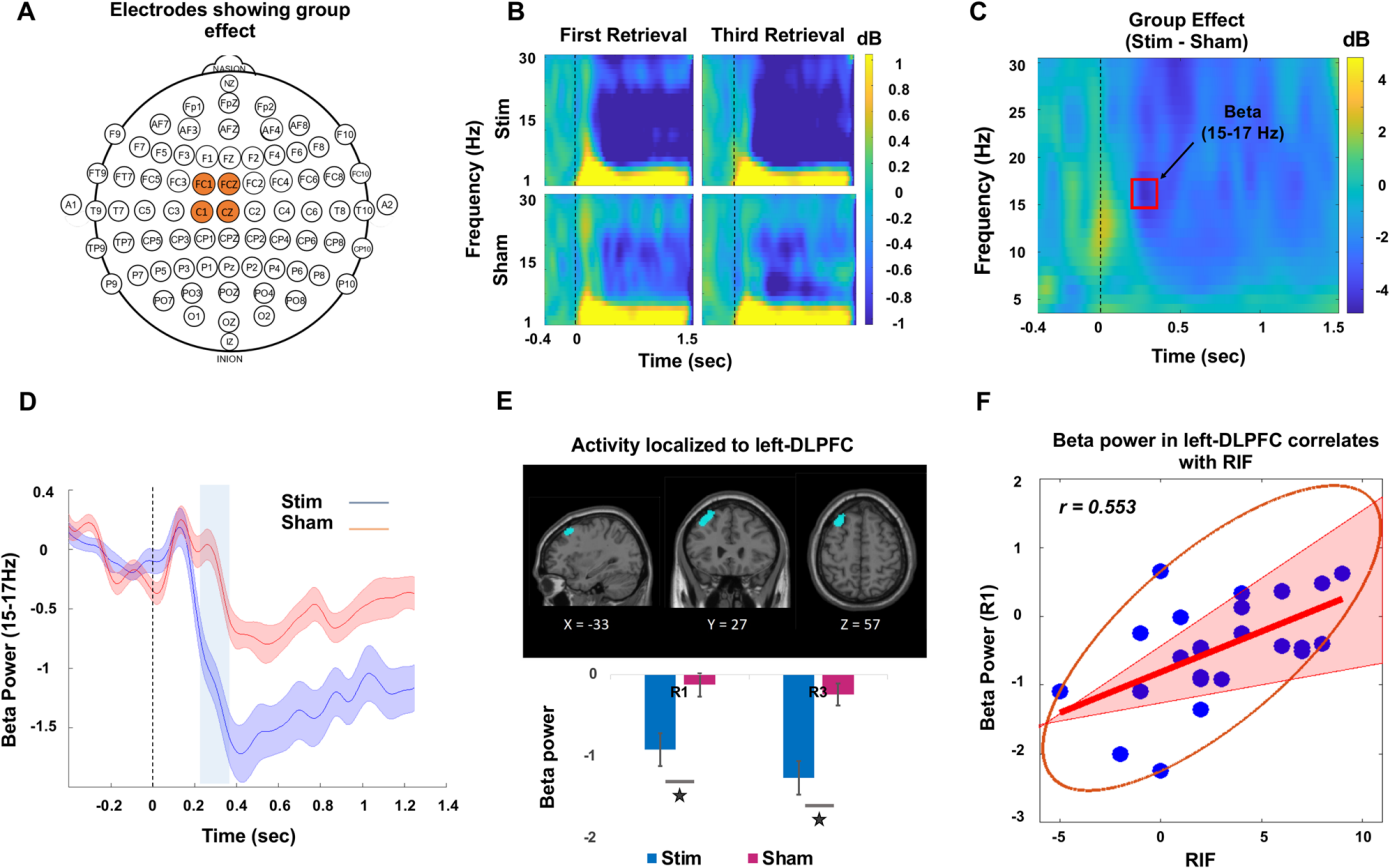
Beta desynchronization in left-DLPFC in an early time window indexes modulation of RIF. ***A***, Electrodes which showed a significant group effect. ***B***, Time–frequency plot for the first and third retrieval sessions from both stimulation and sham groups depicting a stronger beta desynchronization in the stimulated group. ***C***, The group effect assessed by taking the difference between stimulation and sham groups for averaged values of R1 and R3. The red box highlights the region which passed the criteria for statistical significance in the time window 0.24–0.32 s with frequency band ranging from 15 to 17 Hz. ***D***, The averaged power values of R1 and R3 extracted from the 15–17 Hz range in the significant channels for both the groups. The shaded area surrounding the mean line indicates the standard error. The vertical blue highlighted region, on the other hand, highlights the statistically significant between-group differences. ***E***, The observed effect in the specified time window and frequency band was localized to the left-DLPFC and the group differences were primarily driven by stronger beta desynchronization in the stimulation group compared with sham group in both first (R1) and third (R3) retrieval sessions (*p < 0.01, unpaired *t* test between groups). ***F***, Beta desynchronization during first retrieval (R1) was directly related to RIF in the stimulation group. The correlation analysis was performed between RIF and the averaged beta power values across all voxels within the left-DLPFC cluster, as identified by source localization. The red line represents the best linear fit based on skipped Pearson’s correlation, and the pink shaded area represents 95% bootstrapped confidence interval. The data points inside the ellipse represent all the non-outlying data points.

Inhibitory effects are expected to occur shortly after the presentation of the retrieval cue, mainly driven by activity in the lateral and medial prefrontal cortices. Our group comparison of the stimulation and sham groups across the first and third retrieval sessions identified two clusters that met the criteria for significance between the groups and both of these effects occurred within early time windows. The first cluster emerged within a brief poststimulus time window of 0.24–0.32 s in channels FC1, FCz, C1, and Cz, coinciding with the lower beta band frequency range (15–17 Hz), as depicted in Figure 3*A*. A broader beta desynchronization pattern can also be observed in the identified channels following stimulation in both retrieval sessions (Fig. 3*B*), while the cluster surviving significance threshold for group differences (stim vs sham) is highlighted in Figure 3*C*. To further emphasize the observation, Figure 3*D* presents the power values extracted from the 15–17 Hz range in the identified channels, indicating that beta power in the stimulation group remained low after stimulation compared with the sham group while only the highlighted region passed the significance criteria.

The observed group effects in the fronto-central channels support the hypothesis that the stimulation might have interfered with inhibitory mechanisms. To identify the location of this effect, we performed source localization using the TFR information, i.e., time of interest (0.24–0.32 s) and frequency of interest (15–17 Hz) from the first cluster. The source analysis utilized all the channels to identify significant effect in the source space using TFR information which lead to the identification of a significant effect in the left dorsolateral prefrontal cortex (left-DLPFC) as shown in Figure 3*E*. The beta power estimates within the left-DLPFC showed a stronger desynchronization in both retrieval sessions in the stimulation group compared with sham. We then correlated this beta desynchronization from each of the retrieval sessions with the estimates of RIF for both the groups separately. We observed that the amount of RIF in the stimulation group strongly correlated with the beta desynchronization in the left-DLPFC during first retrieval session (skipped Pearson *r*_(23)_ = 0.553; CI = [0.222, 0.782]) as shown in Figure 3*F*, whereas no correlation was observed in the sham group (skipped Pearson *r*_(23)_ = −0.159; CI = [−0.580, 0.381]). Importantly, this beta desynchronization in the left-DLPFC did now show a correlation with the measure of FAC in either stimulation (skipped Pearson *r*_(23)_ = 0.178; CI = [−0.288, 0.381]) or sham groups (skipped Pearson *r*_(23)_ = 0.225; CI = [−0.113, 0.557]). This outcome supports our interpretation that within an early time frame, stimulation may have induced disturbance specifically in inhibitory control mechanisms, with the primary involvement of the left-DLPFC in this disruption. While the correlation between RIF and beta desynchronization was evident during the first retrieval session, it was absent in the third retrieval session in both stimulation (skipped Pearson *r*_(23)_ = −0.071; CI = [−0.334, 0.453]) and sham groups (skipped Pearson *r*_(23)_ = 0.205; CI = [−0.206, 0.555]). The findings suggest that left-DLPFC-driven control mechanisms may be more specifically engaged during first retrieval compared with the third retrieval (see Kuhl et al., 2007 for a similar finding).

### DLPFC modulation predicts parietal beta desynchronization

**Figure 4.**
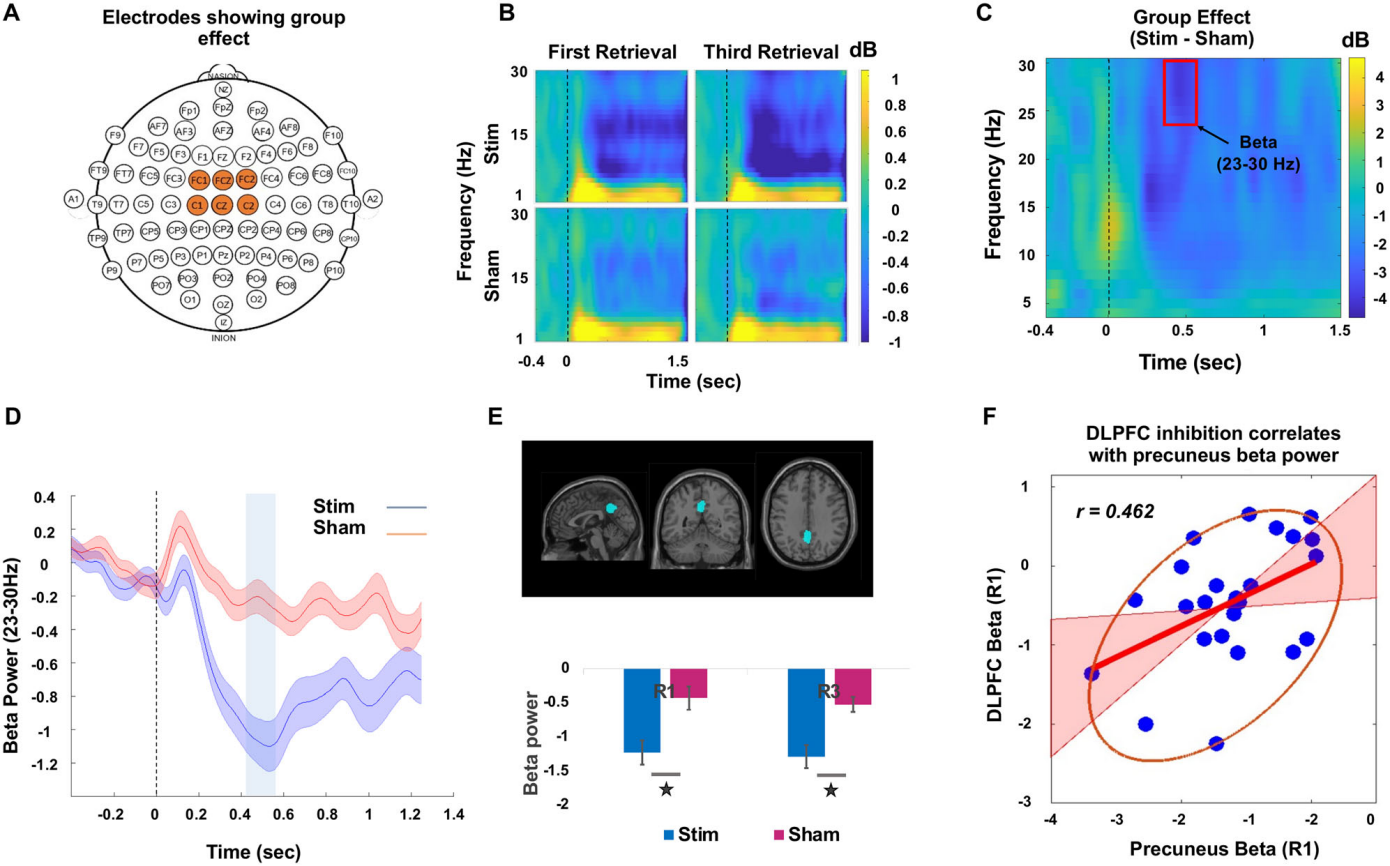
Beta desynchronization in the parietal cortex after stimulation of mPFC. ***A***, Electrodes demonstrating a significant group effect. ***B***, Time–frequency plot for first retrieval (R1) and third retrieval (R3) sessions from both stimulation and sham groups depicting a stronger beta desynchronization in the stimulated group. ***C***, The group effect assessed by taking the difference between stimulation and sham groups for averaged values of R1 and R3 sessions. The red box highlights the region which passed the criteria for statistical significance in the time window 0.41–0.55 s with frequency band ranging from 23 to 30 Hz. ***D***, The averaged power values for R1 and R3 extracted from the 23–30 Hz range in the significant channels for both the groups. The shaded area surrounding the mean line indicates the standard error. The vertical blue highlighted region, on the other hand, highlights the statistically significant between-group differences. ***E***, The observed effect in the specified time window and frequency band was localized to the precuneus cortex, and the group differences were primarily driven by stronger beta desynchronization in the stimulation group compared with sham group in both retrieval sessions (*p < 0.01; unpaired *t* test between groups). ***F***, During first retrieval session, the beta desynchronization in the left-DLPFC was correlated with the beta desynchronization observed in precuneus cortex. The correlation was performed with the averaged data extracted from all the voxels in the clusters in left-DLPFC and precuneus, within the significant time and frequency range. The red line represents the best linear fit based on skipped Pearson’s correlation, and the pink shaded area represents 95% bootstrapped confidence interval. The data points inside the ellipse represent all the non-outlying data points.

The second group effect emerged in the fronto-central channels including FC1, FCz, FC2, C1, Cz, and C2 in the upper beta frequency (23–30 Hz) from 0.41 to 0.55 s poststimulus (Fig. 4*A*,*C*,*D*). Same as cluster one, there was a widespread beta desynchronization in both retrieval sessions as shown in Figure 4*B*. We conducted source localization on the TFRs from the second cluster and identified a source in the precuneus cortex as shown in Figure 4*E*. This effect did not show any correlation with RIF for either of the retrieval sessions and it possibly reflects some other neural processes related to episodic memory retrieval.

Precuneus cortex has often been implicated in episodic memory retrieval processes along with contribution from inferior frontal cortex regions (Lundstrom et al., 2003, 2005). To determine whether the early effects that we observed in beta band in the left-DLPFC relate to the later beta band effects observed in the precuneus cortex, we examined the correlation between these two regions of the brain. We found a significant correlation during the first retrieval session for the stimulation group (skipped Pearson *r*_(23)_ = 0.462; CI = [0.067, 0.717]), as shown in Figure 4*F*. However, this correlation was not observed for the sham group (skipped Pearson *r*_(23)_ = 0.202; CI = [−0.139, 0.509]). A similar but weaker correlation was also observed for the third retrieval session in the stimulation group between these regions (skipped Pearson *r*_(23)_ = 0.360; CI = [−0.041, 0.661]), but not for the sham group (skipped Pearson *r*_(23)_ = 0.112; CI = [−0.296, 0.486]). Although our stimulation protocol was delivered to the prefrontal cortex, the observed effect was located in the precuneus. Moreover, this effect was correlated with an early left-DLPFC effect, suggesting that there is possibly network-level modulation of brain activity, through interactions between the frontal and parietal brain regions. In addition, this parietal effect might relate to memory retrieval processes modulated by disruption of inhibitory control.

**Figure 5.**
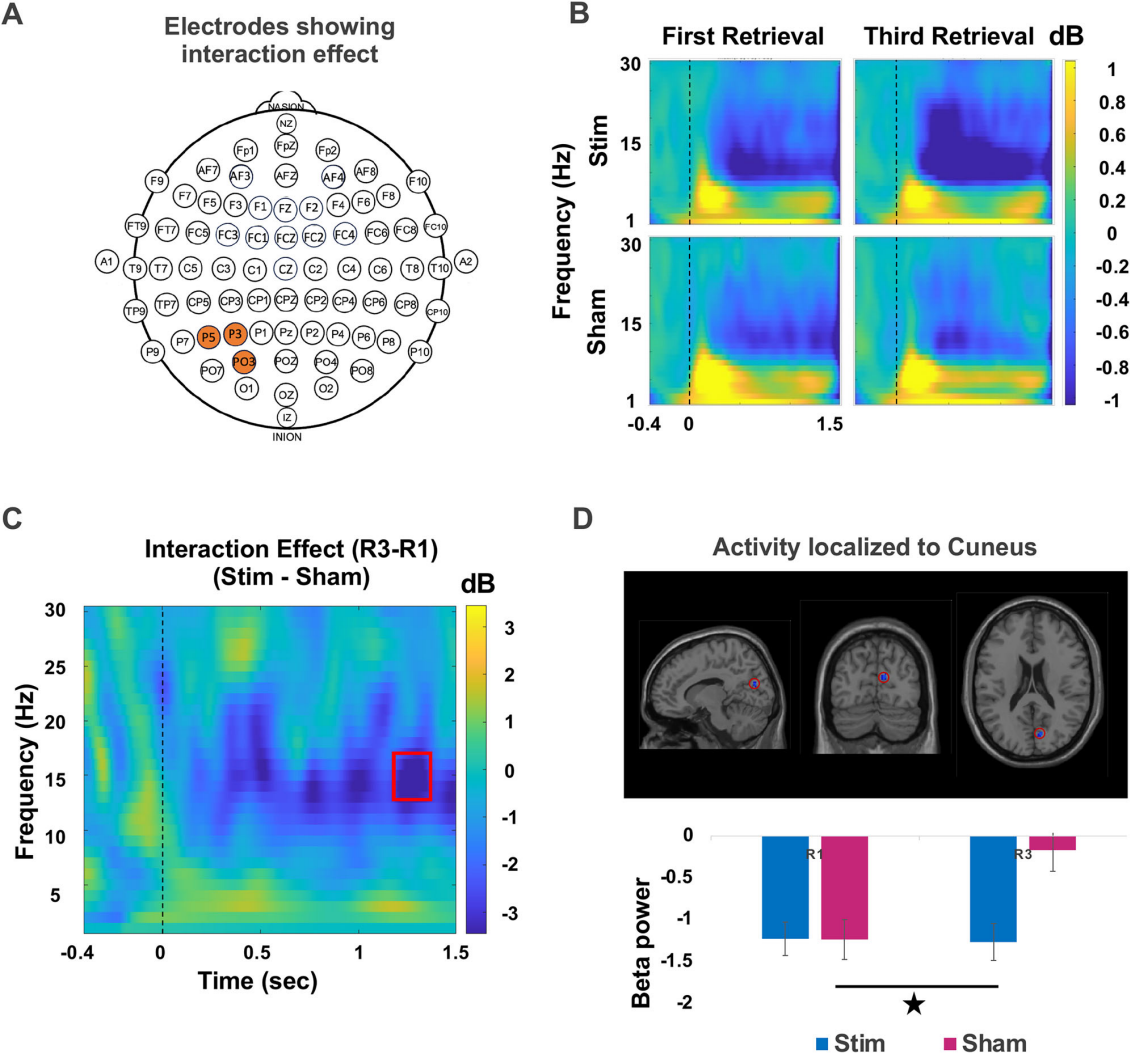
Stimulation led to an increased beta desynchronization in the parietal electrodes. ***A***, Channels which showed a significant interaction effect. ***B***, Time–frequency representations from the first retrieval (R1) and third retrieval (R3) sessions are shown for both the stimulation and sham groups. ***C***, A time–frequency plot illustrating the interaction effect assessed by taking the group difference (stim–sham) between the difference in the activity of R1 and R3 sessions, with significant differences highlighted in the red box. ***D***, The source activity of the interaction effect was localized to the cuneus. The stimulation group showed a sustained beta power across retrieval sessions, in contrast to the declining trend observed in the sham group (*p < 0.01, ANOVA between groups for R1 and R3).

Finally, we investigated the interaction effect by applying permutation testing on the difference of activity between first and third retrieval session. We identified a significant cluster in the parietal channels including P3, P5, and PO3 in the frequency range 14–16 Hz and time window of 1.256–1.328 s (Fig. 5*A–C*). Source localization conducted on the interaction effect TFR window localized the effect to cuneus and this interaction effect was mainly driven by a sustained beta desynchronization in the stimulation group; however, the beta power dropped during the third retrieval in the sham group as shown in Figure 5*D*. The beta activity for either of the retrieval sessions for each group did not correlate with RIF and neither with the beta desynchronization observed in the left-DLPFC.

Notably, RIF did not show any correlation with any of the effects observed in the parietal cortex. This finding reinforces the hypothesis that inhibitory control mechanisms are primarily mediated by the prefrontal cortex, while modulation of the parietal brain regions may reflect other cognitive effects related to memory retrieval, as discussed in detail in the Discussion section. In addition, we would like to emphasize that there was a widespread beta desynchronization in the fronto-central and parietal brain regions following stimulation. The identified group and interaction effects in very specific windows appeared only after applying strict thresholding criteria.

### Conflict reduction benefit

One a priori hypothesis we had was that stimulation would interfere with inhibitory control by modulating theta band activity. This was based on previous findings where a decrease in theta band activity over repeated retrievals reflected the amount of RIF (Hanslmayr et al., 2010), often termed as conflict reduction benefit. Despite this, we did not observe any significant group or interaction effects in theta band activity.

**Figure 6.**
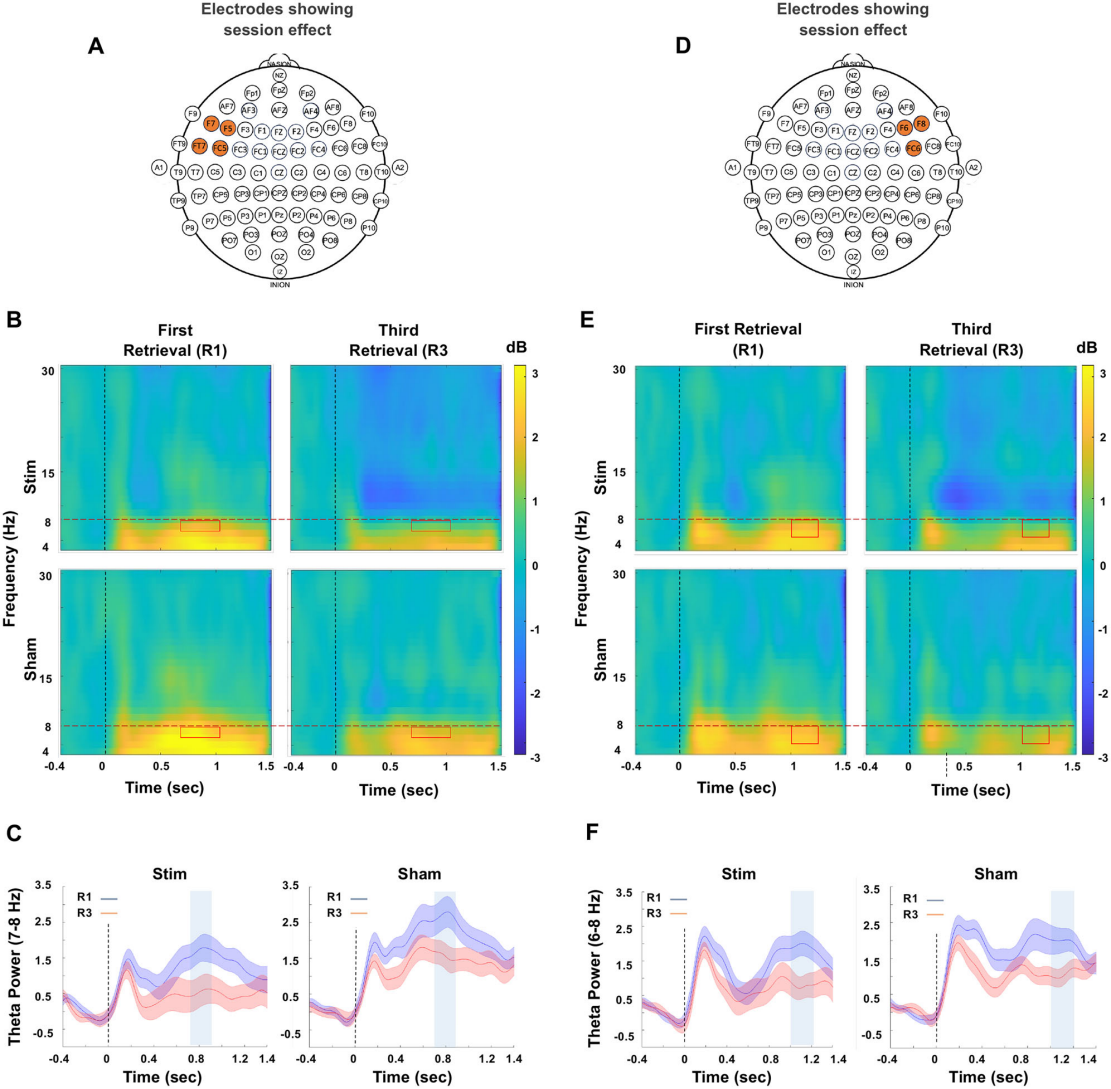
Conflict reduction benefit. ***A***, ***D***, The channels that exhibited significant session-related effects. ***B***, The time–frequency plots of the first and third retrieval sessions for the left frontal cluster, while ***E*** shows it for right frontal cluster with significant session effect highlighted in red box. The analysis was restricted to theta band (4–8 Hz), as indicated by the red horizontal dotted line. ***C***, ***F***, The TFR extracted from the identified clusters within the theta frequency band in the left and right frontal electrodes, respectively. Participants in both groups demonstrated a statistically significant decline in theta power, in the highlighted blue region, during the third retrieval compared with the first in both left and right frontal electrodes.

We explored the possibility that the absence of modulation in the theta band could be due to the overpowering effect of beta band activity on permutation testing. To further investigate, we conducted an additional permutation test to assess group, session, and interaction effects specifically within the theta band frequency range (4–8 Hz). Our analysis did not reveal any significant clusters that met the criteria for significance for group and interaction effects (*p* < 0.001). However, we did observe session effects (estimated by combining stimulation and sham groups together and running permutation test on R1 and R3) in the theta band which showed a decrease in theta power in R3 as compared with R1. Session effects appeared in electrodes positioned over both the left and right frontal regions of the brain. The initial effect became apparent within the time span of 0.7210–1.0840 s after the onset of the event, specifically in left frontal channels F5, F7, FC5, and FT7 in 7–8 Hz range (Fig. 6*A*). Subsequently, the second effect was observed between 1.0280 and 1.2480 s, in right frontal channels F6, F8, and FC6 in 6–8 Hz range (Fig. 6*D*). The time–frequency plots for both first and third retrieval sessions for both the groups are shown in Figure 6*B*,*E* for the left frontal and right frontal channels, respectively. The theta power extracted from the identified left frontal and right frontal clusters is shown in Figure 6*C*,*F*, respectively, which shows a decrease in theta power in third retrieval compared with first retrieval while the highlighted region shows the area which passed the criteria for statistical significance. However, the decrease in theta power over repeated retrievals, estimated by taking the difference between the third and first retrieval effects, did not show a correlation with RIF for either left frontal clusters (stim: skipped Pearson *r*_(23)_ = 0.179, CI = [−0.159, 0.523]; sham: skipped Pearson *r*_(23)_ = −0.206, CI = [−0.117, 0.487]) or right frontal clusters (stim: skipped Pearson *r*_(23)_ = −0.326, CI = [−0.756, 0.138]; sham: skipped Pearson *r*_(23)_ = 0.218, CI = [−0.133, 0.545]).

### Stimulation influence goes beyond memory

**Figure 7.**
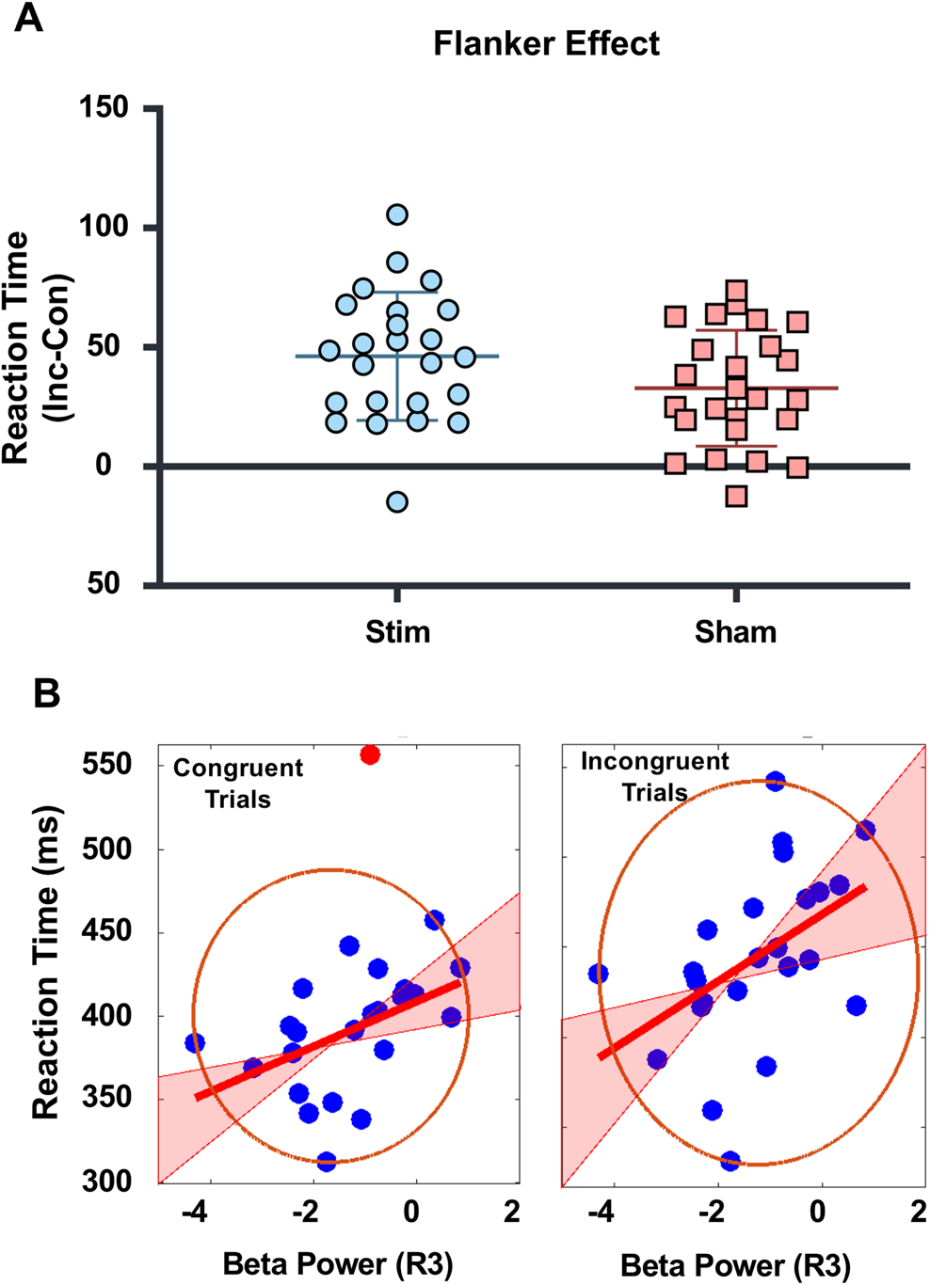
Stimulation impacted the performance on the Flanker task, which was administered as a distractor task immediately after retrieval practice phase. ***A***, The stimulated group showed weaker ability to resist distractions compared with the sham group. The figure shows the flanker effect estimated by taking the difference of reaction time between the incongruent and congruent trials with stimulation group showing a larger flanker effect than shown by the sham group. ***B***, The reaction time for congruent trials and incongruent trials in the stimulation group correlated with beta band activity during third retrieval practice (R3), with more beta desynchronization predicting faster reaction time. The red line represents the best linear fit based on skipped Pearson’s correlation, and the pink shaded area represents 95% bootstrapped confidence interval. The data points inside the ellipse represent all the non-outlying data points.

The study employed a flanker task, a classic measure of attentional control, as a distractor task administered after the retrieval practice phase. The task involves participants responding to a target stimulus while ignoring conflicting cues (i.e., flankers) that surround it. A stronger flanker effect, estimated by taking the difference between reaction time of incongruent and congruent trials, indicates greater interference from the distractors, suggesting impaired inhibitory control. In the flanker task, one participant's data was excluded because the event codes were not recorded. This left us with data from 49 participants (24 in the stimulation group and 25 in the sham group) for analysis. Our study found a marginally significant difference in the flanker interference effect between the stimulation and sham groups (*t*_(47)_ = 1.83; *p* = 0.074). Participants in the stimulation group exhibited a more pronounced flanker effect (mean = 46.17; SD = 26.85) compared with those in the sham group (mean = 32.80; SD = 24.30) as shown in Figure 7*A*. This finding, while not the primary focus of our investigation, provides evidence that mPFC stimulation disrupts inhibitory control, possibly extending beyond memory-related tasks. In addition, the weak influence of stimulation on the flanker task could be explained by a gradual decline in stimulation impact over time.

To further investigate the hypothesis that a common inhibitory control process may be involved in RIF and flanker task, we correlated the beta band desynchronization observed during the retrieval of memories with the performance on flanker task. We restricted our analysis to the first statistically significant cluster identified in the memory retrieval task that also correlated with performance on the RIF task reflecting modulation of inhibitory control. However, the correlation analysis between retrieval beta and flanker effect did not show any significant correlation for either first (skipped Pearson *r*_(22)_ = 0.225; CI = [−0.155, 0.574]) or third (skipped Pearson *r*_(22)_ = 0.152; CI = [−0.219, 0.541]) retrieval sessions for the stimulation group. Surprisingly, however, the beta band activity during the third retrieval predicted the reaction time in congruent trials (skipped Pearson *r*_(22)_ = 0.478; CI = [0.241, 0.699]) and incongruent trials (skipped Pearson *r*_(22)_ = 0.462; CI = [0.229, 0.707]), with stronger beta desynchronization reflecting faster reaction time in both the trial types in the stimulation group as shown in Figure 7*B*. Notably, these correlations were not observed in the sham group for either the congruent (skipped Pearson *r*_(23)_ = 0.160; CI = [−0.472, 0.246]) or incongruent (skipped Pearson *r*_(23)_ = −0.162; CI = [−0.414, 0.161]) trials. Further analysis of reaction times revealed an interesting pattern. While the flanker effect was marginally significant between groups, there were no significant differences between groups in how fast they responded to either congruent (*t*_(47)_ = 1.58; *p* = 0.253) or incongruent (*t*_(47)_ = 0.360; *p* = 0.721) trials. This suggests that some underlying mechanisms in both congruent and incongruent trials were altered in a way that increased the flanker effect. Interestingly, only beta band activity from the retrieval session closest to the flanker task seemed to predict the reaction time for both the trial types. While our results showed a link between beta band activity during retrieval and reaction times in the flanker task, they do not directly confirm that this activity reflects the flanker effect itself. Importantly, on a behavioral level, there is currently no existing research directly demonstrating a relationship between RIF and the flanker effect. Our findings also do not provide evidence for a relationship between RIF and the flanker effect for either stimulation (skipped Pearson *r*_(22)_ = −0.0518; CI = [−0.367, 0.407]) or the sham group (skipped Pearson *r*_(23)_ = −0.096; CI = [−0.508, 0.266]). Nevertheless, our findings show that beta desynchronization during memory retrieval is associated with both changes in RIF and flanker task performance. However, the precise mechanism with which it influences flanker task performance needs further investigation.

We further analyzed the accuracy data from flanker task to see if stimulation had any influence on difference in error rate for congruent and incongruent trials. However, no stimulation-related effect was observed between groups for the flanker effect on accuracy (*t*_(47)_ = 0.879; *p* = 0.384).

### Stimulation-related side effects

No stimulation-related side effects were observed for any of the measured variables assessed using unpaired *t* test, including headache (*t*_(48)_ = 0.34; *p* = 0.561), neck pain (*t*_(48)_ = 0.34; *p* = 0.561), scalp pain (*t*_(48)_ = 1.75; *p* = 0.192), tingling (*t*_(48)_ = 0.18; *p* = 0.677), itching (*t*_(48)_ = 2.79; *p* = 0.102), burning sensation (*t*_(48)_ = 0.28; *p* = 0.602), sleepiness (*t*_(48)_ = 0.23; *p* = 0.631), concentration (*t*_(48)_ = 2.24; *p* = 0.141), and mood (*t*_(48)_ = 0.00; *p* = 1.000). One participant reported feeling tired after the experiment. Participants were not explicitly asked about their group assignment after the experiment. However, the stimulation and sham protocols used in the study are consistent with the standard stimulation protocols (Woods et al., 2016). Moreover, the absence of differences in their subjective feelings, as assessed by the post-experiment questionnaire, supports the assumption that the subjective effects of the stimulation and sham conditions were similar.

## Discussion

In this study, we investigated the causal role of the mPFC in RIF, a measure of inhibitory control, by stimulating the mPFC during memory retrieval within a RIF paradigm. Additionally, we examined the electrophysiological factors underlying these effects. Our primary findings reveal that stimulation of mPFC prior to retrieval practice selectively reduced the amount of RIF observed during the final test phase, without affecting FAC. Electrophysiological data showed that stimulation induced stronger beta desynchronization in the fronto-parietal brain regions. Particularly, the effect observed from 0.24 to 0.32 s after cue onset, originating from the left-DLPFC, predicted the amount of RIF during the later test phase. These findings indicate that mPFC stimulation reduces RIF and increases beta desynchronization in the fronto-parietal brain regions, possibly reflecting modulation of inhibitory control processes by stimulation.

As expected, consistent with prior work, we found that retrieval practice of target memories induces forgetting of non-target competitive memories (Anderson et al., 1994; Hanslmayr et al., 2010; Staudigl et al., 2010; see Anderson and Hulbert, 2020; Marsh and Anderson, 2022 for reviews). Importantly, however, we demonstrate that stimulating mPFC prior to selective retrieval practice decreases RIF compared with that observed in a group receiving sham stimulation. Reduced RIF was mainly driven by higher final recall for non-target competing items (RP− items), indicating that stimulation of the mPFC during retrieval practice reduced the tendency to forget these key items on the delayed test. This finding is consistent with the possibility that stimulating mPFC disrupted some aspect of an inhibitory control process reliant on the mPFC, preventing inhibition from suppressing competing items during retrieval practice and reducing RIF. Disrupted RIF was observed despite comparable performance in retrieving target items during the final test recall (FAC) across the stimulation and sham groups and despite both groups showing improved retrieval practice accuracy over repetitions during retrieval practice phase. Supporting the inhibitory model of RIF, our data indicate that forgetting of competitors arises as a result of retrieving target memories and that these two processes are functionally independent of each other.

Other psychological manipulations have also been found to selectively disrupt RIF, without affecting FAC or retrieval practice success. For example, inducing stress before retrieval practice selectively abolished RIF with no effect on retrieval practice accuracy or FAC during the final memory recall (Koessler et al., 2009). A study by Kuhbandner et al. reported that inducing negative moods abolished RIF, whereas inducing positive and neutral moods had no impact on RIF (Bäuml and Kuhbandner, 2007). It is important to note that the participants in our study did not report any mood alterations following stimulation, indicating that the observed RIF effect is not associated to mood changes. Furthermore, performing a divided attention task during retrieval practice also selectively disrupted RIF during the final test (Román et al., 2009; Ortega et al., 2012; Mulligan et al., 2022). Selective disruption of RIF has also been observed after stimulation of right-DLPFC (Penolazzi et al., 2014; Stramaccia et al., 2017; Valle et al., 2020), consistent with the possibility that disruption of this region compromised inhibitory control.

Additionally, theta power has been traditionally associated with cognitive processing, and midfrontal theta is recognized as an important marker for inhibitory control in attention- and memory-related contexts (Hanslmayr et al., 2010; Staudigl et al., 2010; Kim et al., 2014). In the context of RIF, it has been demonstrated that larger decreases in theta power over repeated retrieval practice trials predicts increased RIF observed in a subsequent test, suggesting decreased cognitive demand with repeated practice, often termed as conflict reduction benefit (Hanslmayr et al., 2010; see Anderson and Hulbert, 2020 for a review). Replicating prior work, here we observed a significant decrease in theta power in the third retrieval practice compared with the first retrieval in left and right prefrontal regions in both the real and sham stimulation groups. Unexpectedly, however, stimulating the mPFC with direct current did not affect overall theta power observed during retrieval practice, nor did it affect the decline in theta over practice trials. Thus, direct current stimulation before retrieval had no measurable impact on midfrontal theta activity during the retrieval task. Given this observation, the modulation of RIF observed in our study more likely derives from its impact on other neural processes not mediated by theta activity.

The other neural marker in the frontal cortex which has often been associated with inhibition is beta band activity (Hwang et al., 2014; Castiglione et al., 2019; Hubbard and Sahakyan, 2023). In our study, we observed a stronger beta desynchronization in the fronto-central channels in the stimulation group compared with the sham group during retrieval sessions. These effects were localized in the left-DLPFC and precuneus cortex. Importantly, the enhanced beta desynchronization in the left-DLPFC correlated significantly with RIF in the stimulation group, indicating that stronger beta desynchronization may indicate greater disruption of inhibitory control. In the context of inhibitory control, the timing of the occurrence of this effect is crucial. This modulation is consistent with earlier research indicating that inhibition occurs early in the memory retrieval process, with the involvement of DLPFC regions (Castiglione et al., 2019; Crespo-García et al., 2022). However, we found no correlation between beta activity and RIF in the sham group, an observation that at first seems inconsistent with the hypothesis that beta reflects inhibitory control. However, it is possible that the brain recruits additional resources to compensate for the effect of stimulation and that reflected only in the stimulated group in the form of stronger beta desynchronization.

While increased neural synchronization has been traditionally associated with successful memory retrieval, decreased synchronization in specific frequency bands, particularly the alpha and beta bands within the parietal cortex, is also considered a hallmark of successful memory retrieval (Spitzer et al., 2008; Hanslmayr et al., 2012). Surprisingly, we observed a strong beta desynchronization in the parietal cortex in the stimulation group, and this effect was source localized to the precuneus. Such modulation of the activity in the parietal cortex, possibly through network level modulation, has also been reported in several other studies (van der Plas et al., 2021; Khan et al., 2023). Considering the observations presented above, the increased beta desynchronization in the precuneus could either reflect greater retrieval of retrieval practice targets or, alternatively, increased retrieval of competitors. Given that retrieval practice performance did not vary between the stimulation and sham groups, we have little overt behavioral indication that stimulation led to more target retrieval. We do, however, have behavioral evidence that stimulation reduced RIF by selectively increasing later recall of competing items, suggesting that competitors were less likely to be inhibited by retrieval of target items. Thus, the increased beta desynchronization in the parietal cortex may reflect an increase in retrieval of non-target memories during retrieval practice, resulting from a disruption of inhibitory control in the prefrontal cortex induced by stimulation.

Whereas our study shows that stimulating mPFC with anodal stimulation may have disrupted inhibitory control, other studies have reported contradictory findings. For instance, online anodal stimulation applied to the mPFC is reported to improve inhibitory control in an attention task (To et al., 2018; Khan et al., 2022). This inconsistency might stem from stimulation timing, as online and offline stimulation can have dramatically different effects (Stagg and Nitsche, 2011; Pirulli et al., 2013). Alternatively, the inhibitory control in attention and memory might engage distinct cognitive processes that respond differently to stimulation. Nevertheless, the stimulation also increased flanker interference effect, suggesting modulation of inhibition in an attention task. However, the observed increase in the Flanker effect was not predicted by either RIF or by beta modulation observed during preceding memory retrieval. Interestingly, both congruent and incongruent reaction times correlated with beta desynchronization during preceding memory retrieval. These findings imply that stimulation may have influenced common inhibitory control processes involved in both attention-demanding tasks and memory inhibition. A recent review underscores beta band activity in the frontal cortex as a promising marker of inhibitory control across domains (Wessel and Anderson, 2023). Nonetheless, further investigation is necessary to fully understand how stimulation affects these common inhibitory processes.

In conclusion, the study demonstrates that mPFC stimulation prior to memory retrieval selectively interferes with the processes responsible for RIF, as assessed in a subsequent test phase. We further show that while forgetting occurs as a result of retrieving certain memories, both processes are functionally independent of each other. Importantly, the decrease in RIF was associated with a more pronounced desynchronization of beta band activity (15–17 Hz) in the left-DLPFC in an early time window (0.24–0.32 s) during retrieval practice trials. In a later time window, the stimulation group exhibited sustained stronger beta desynchronization in the parietal cortex, possibly reflecting unintended retrieval of competing memories. Together, our findings suggest that stimulation-induced beta desynchronization in the fronto-parietal cortex may reflect disrupted inhibitory control mechanisms that reduced RIF. However, the mechanisms by which the mPFC contributes to inhibitory control processes need further exploration.
